# Genomic scanning enabling discovery of a new antibacterial bicyclic carbamate-containing alkaloid

**DOI:** 10.1016/j.synbio.2021.01.002

**Published:** 2021-01-20

**Authors:** Qing Fang, Linrui Wu, Caroline Urwald, Morgane Mugat, Shan Wang, Kwaku Kyeremeh, Carol Philips, Samantha Law, Yongjun Zhou, Hai Deng

**Affiliations:** a Marine Biodiscovery Centre, Department of Chemistry, University of Aberdeen, Meston Walk, Aberdeen AB24 3UE, Scotland, UK; b ENSAIA, 2 avenue de la forêt de Haye, 54505 vandœuvre lès Nancy, France; c Department of Chemistry, University of Ghana, P.O. Box LG56, Legon-Accra, Ghana; d NCIMB Ltd, Ferguson Building, Craibstone Estate, Bucksburn, Aberdeen, AB21 9YA, Scotland, UK; e Research Centre for Marine Drugs, State Key Laboratory of Oncogenes and Related Genes, Department of Pharmacy, Ren Ji Hospital, School of Medicine, Shanghai Jiao Tong University, Shanghai, 200127, China

**Keywords:** Genomic scanning, Bayer villiger monooxygenase, Carbamate alkaloids, Pyrrolizidine alkaloids, Non-ribosomal peptide synthetases

## Abstract

Non-ribosomal peptides are a group of structurally diverse natural products with various important therapeutic and agrochemical applications. Bacterial pyrrolizidine alkaloids (PAs), containing a scaffold of two fused five-membered ring system with a nitrogen atom at the bridgehead, have been found to originate from a multidomain non-ribosomal peptide synthetase to generate indolizidine intermediates, followed by multistep oxidation, catalysed by single Bayer-Villiger (BV) enzymes, to yield PA scaffolds. Although bacterial PAs are rare in natural product inventory, bioinformatics analysis suggested that the biosynthetic gene clusters (BGCs) that are likely to be responsible for the production of PA-like metabolites are widely distributed in bacterial genomes. However, most of the strains containing PA-like BGCs are not deposited in the public domain, therefore preventing further assessment of the chemical spaces of this group of bioactive metabolites. Here, we report a genomic scanning strategy to assess the potential of PA metabolites production in our culture collection without prior knowledge of genome information. Among the strains tested, we found fifteen contain the key BV enzymes that are likely to be involved in the last step of PA ring formation. Subsequently one-strain-many-compound (OSMAC) method, supported by a combination of HR-MS, NMR, SMART 2.0 technology, and GNPS analysis, allowed identification and characterization of a new [5 + 7] heterobicyclic carbamate, legoncarbamate, together with five known PAs, bohemamine derivatives, from *Streptomyces* sp. CT37, a Ghanaian soil isolate. The absolute stereochemistry of legoncarbamate was determined by comparison of measured and calculated ECD spectra. Legoncarbamate displays antibacterial activity against *E. coli* ATCC 25922 with an MIC value of 3.1 μg/mL. Finally, a biosynthetic model of legoncarbamate and other bohemamines was proposed based on the knowledge we have gained so far.

## 1 Introduction

**Fig. 1:**
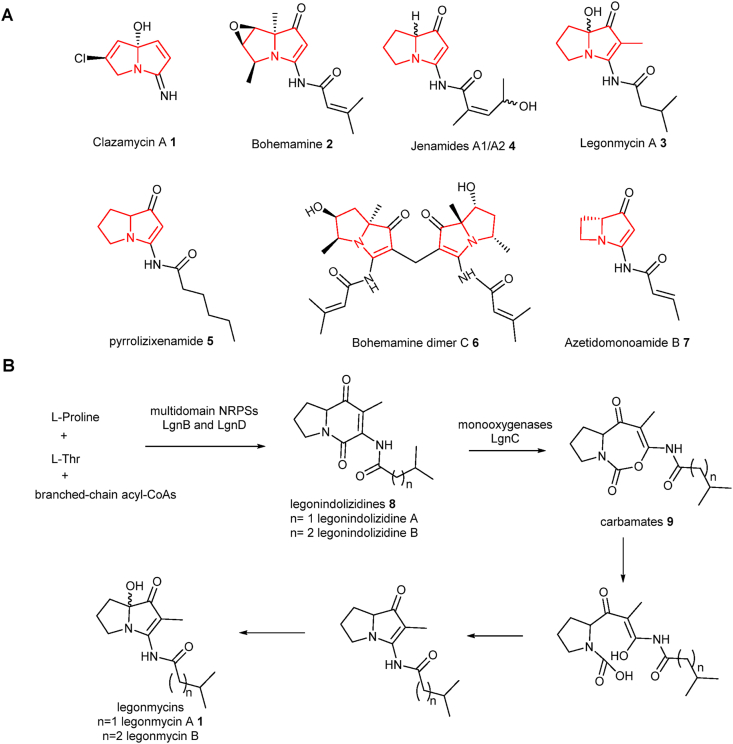
**A**. Representatives of bacterial pyrrolizidine alkaloids. **B**. A proposed biosynthetic model of legonmycins.

Pyrrolizidines are a group of organic compounds that possess a fused bicyclic five-membered rings with a nitrogen atom at the bridgehead position. While pyrrolizidine alkaloids (PAs) have been mainly found as plant metabolites, some of which are part of plant defence mechanism against insect herbivores, less than 30 PAs have been discovered from bacterial origins [1]. The representatives include clazamycins **1** [2], bohemamine **2** [3], jenamides A1/A2 **4** [4], and more recently legonmycins **3** [5], pyrrolizixenamide **5** [6], bohemamine dimer C **6** [7], and azetidomonamide (azabicyclene) **7** [8,9] (Fig. 1A). Bacterial PAs exhibit a broad range of bioactivities and as such they have attracted considerable interest from both academic research groups and the pharmaceutical industry [11].

Recently we disclosed the biosynthetic origin of two bacterial PA specialised metabolites (SMs), denoted as legonmycins A and B [4], isolated from a talented Ghanaian isolate, *Streptomyces* sp. MA37 [4,[10], [11], [12], [13], [14], [15], [16]]. We have demonstrated that legonmycins are biosynthesized from an NRPS assembly line to generate the key intermediates, legonindolizidines **8** ([5 + 6] heterobicyclic ring system). The monooxygenase LgnC acts on **8** and catalyses a multistep Baeyer-Villiger process of ring expansion to afford carbamates, **9** ([5 + 7] heterobicyclic ring system). Subsequent ring-opening by hydrolysis and decarboxylation-driven ring contraction finally provides the [5 + 5] heterobicyclic pyrrolizidine ring (Fig. 1B) [4].

The biosynthetic gene clusters (BGCs) that are likely to be responsible for PA productions have been shown to be widely spread in bacterial genomes, suggesting that PA SMs may have important biological functions [6]. One of the key features among these BGCs is that they all encode LgnC-like monooxygenase open reading frames (ORFs) that are likely to be responsible for the biotransformation from [5 + 6] indolizidine intermediates to [5 + 5] PA scaffolds. Bioinformatics analysis of these putative BGCs indicated that the biosynthetic genes encoding tailoring catalytic functions appear to be diverse [6]. In contrast to the biodiversity of BGCs, the chemical space of this group of bacterial PA SMs has not been fully assessed. Many of these bacterial strains are not deposited in the public domain. Thus, it is difficult to assess whether these strains are able to produce PA-like metabolites with chemical diversity under laboratory conditions. Considering that LgnC-type monooxygenases are the key enzymes to biosynthesize PA metabolites, we reasoned that it was possible to apply the PCR-based genomic scanning strategy of using the sequences that are conserved among LgnC-type monooxygenases as probes to identify strains in either public or our own culture collections that may contain PA BGCs, followed by facilitating one strain many compounds (OSMAC) method [17] to isolate new PA metabolites for biological assessments.

Here we report the application of PCR-based genomic scanning of 58 actinomycete strains from NCIMB, the largest bacterial culture collection in the UK and our own culture collection. This resulted in the identification of 15 potential PA producing strains. Among these strains, one of our own cultures, the Ghanaian isolate *Streptomyces* sp. CT37 (CT37) was found to produce PA-like metabolites. Subsequent chemical workup and structural elucidation allowed the identification of an unusual [5 + 7] heterobicyclic carbamate alkaloid, legoncarbamate **10**, and a known PA metabolite, NP25302 **11**. NOE analysis together with electronic circular dichroism (ECD) measurement and computational calculation confirmed that **10** has (5*S*, 8*S*) configuration. Further analysis of the semi-purified extracts from the culture of CT37 using LC-MS-based Global Natural Products Social molecular networking (GNPS) [18], in conjunction with newly developed NMR-based machine learning tool “Small Molecule Accurate Recognition Technology” (SMART 2.0) [19] allowed identification of the presence of four known bohemamine SMs **15**–**18**. *In silico* analysis of the annotated genome of CT37 together with current knowledge of PA biosynthesis allowed identification of the BGC of the carbamate alkaloid **10** and other PA-related SMs **15**–**18**.

## 2 Results and discussion

With recently discovered PA metabolites and their corresponding BGCs, we compared the biosynthetic enzymes encoded in these BGCs with key legonmycin biosynthetic enzymes. It appears that this group of SMs are assembled by multidomain non-ribosomal peptide synthetases (NRPSs) to generate indolizidine intermediates, followed by essential LgnC-like monooxygenases to provide PA bicyclic frameworks. LgnC-like monooxygenases catalyse unusual multistep chemical transformation from indolizidine via carbamate to pyrrolizidine [4] which is clearly different from other Bayer-Villiger (BV) enzymes [20]. Therefore, it is possible to apply degenerate primers, according to the conserved amino acids sequences of these monooxygenases, to scan genomic DNAs of a large amount of available bacterial collections in order to identify potential PA producing strains. To this end, we adopted a bioinformatics-based approach to break the trend in these BV monooxygenases (Fig. S1A). Having compiled data from reported putative BV monooxygenase enzymes together with other types of BV monooxygenases, protein motif elicitor (MEME) [21] analysis enabled us to identify three highly conserved motifs: GxGxxG, YWWxTKN, and GDAAH (where ‘x’ is any amino acid and each independently represents the number of x between each conserved residue) (Fig. S1A). GxGxxxG motif has hitherto been used to indicate the presence of FAD which could as readily indicate flavin-monooxygenase-related enzymes [22]. However, YWWxTKN, and GDAAH motifs, covering approximate 80 amino acid residues, are unique among LgnC-like monooxygenases and as such these two motifs may be used as PCR probe to screen LgnC-like monooxygenase from uncurated genomic DNAs of available bacterial strains (Table S1).

To examine the feasibility of PCR-based genomic scanning approach, we first amplified the internal fragment of *lgn*C with our designed degenerate primers using the genomic DNA of *Streptomyces* sp. MA37 as the positive control and *Streptomyces albus* as a negative control. The expected PCR product with approximately 240 bp, were observed in DNA electrophoresis analysis (Fig. S1B) in the positive control but not in the negative control. The DNA sequence of the PCR product was further confirmed to be the targeted internal DNA fragment of *lgn*C by DNA sequencing.

In the collaborative natural product discovery programme with NCIMB Ltd, we obtained 56 *Streptomyces* strains (Table S2). We extract the genomic DNAs from these strains together with soil isolates in our laboratory including the new indole alkaloid SM producer, *Streptomyces* sp. CT37 (CT37) [23], and performed PCR scanning using these DNAs as templates. Among these strains, we were able to identify fifteen positive PCR results with the expected DNA length. These DNAs were then cloned into a commercially available plasmid via TA cloning method (Method and experiments). The constructs were extracted from the positive clones which was subjected for DNA sequencing. Finally, the resulting DNA sequences were annotated using FramePlot to predict protein-coding regions. The amino acid alignment with the one in LgnC as a control and phylogenetic analysis indicated that these amplified DNAs are indeed encoded the fragments (~80 amino acid (AA) residues) of LgnC-like monooxygenases with high AA similarity (80%–95%), suggesting that these strains potentially possess these BV monooxygenases (Fig. S2).

In order to examine whether these strains can produce PA-like metabolites under laboratory conditions, we applied the OSMAC approach to activate the cryptic PA-like BGCs among these strains. Eight standard *Streptomyces* media (ISP2, Modified Bernett's, ISP3, ISP4, ISP5, ISP6, ISP7, Starch Casein) available in our laboratory were used (Table S3). After small scale fermentation (50 mL, 7 days, 28 °C), one hundred twenty of these culture broths were extracted using ethyl acetate to generate crude extracts, followed by LC-HRESIMS analysis. Targeted metabolomics using GNPS network analysis were then applied to construct extensive metabolite networks of these extracts. Detailed analysis of these networks enabled the identification of a small node network from an extract of CT37 that is likely to contain PA-like metabolites (Fig. S3). With this information on hands, we carried out a large fermentation (8 L) of CT37 in MB medium (7 days, 28 °C). To absorb small molecule components, Diaion®HP-20 (3 g/50 mL, Mitsubishi Chemical Co., Binasco, Italy) was added. After filtration, resins were extracted extensively with methanol to generate crude extracts, followed by vacuum liquid chromatography to provide ten fractions. Fractions containing PA-like metabolites were combined and subjected to semipreparative HPLC separation to yield a new compound, **10** (1.0 mg), together with one known bohemamine metabolite, NP25302 **11** (1.5 mg) [3], and two semi-purified fractions, each of which is likely to contain a mixture of two analogue compounds that were proved difficult to be separated.

**Fig. 2:**
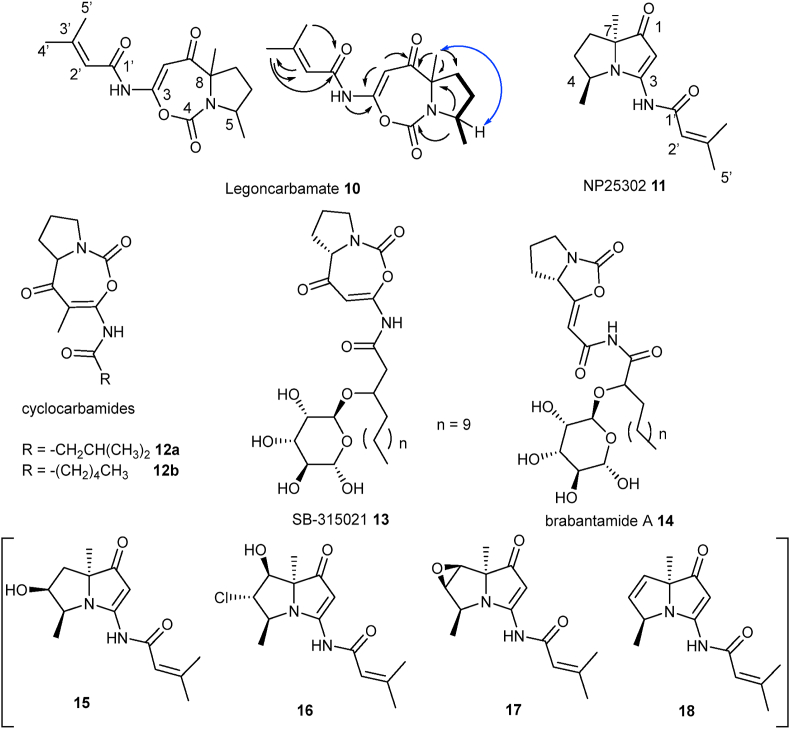
Structures of legoncarbamate **10** and NP25302 **11** together with two known metabolites that contain [5 + 7] carbamate ring systems, cyclocarbamide **12**, SB315021 **13** and the metabolite brabantamide A **14**. Compound **10** with key correlation spectroscopy (COSY) (−), key heteronuclear multiple bond correlation (HMBC) (→) and NOE (→) correlations. The structures of compounds **15**–**18** [3,24,25] in the bracket were deduced from the analyses of NMR-based artificial intelligence SMART 2.0 technology [26] in conjunction with LC-MS-based GNPS [18] and genomic context.

**10** was obtained as a white powder with a molecular formula of C_15_H_20_N_2_O_4_^+^ deduced by high-resolution electrospray ionization mass spectrometry (HRESIMS) (observed *m/z* [M+H]^+^ = 293.1502, calculated [M+H]^+^ = 293.1496; Δ = 2.047 ppm) with 7° of unsaturation (Figs. S4–5). The planar structure of **10** was characterized using spectrometric and spectroscopic analyses including HRESIMS, 1D and 2D nuclear magnetic resonance spectroscopy (NMR) (Fig. 2, Table S4, Figs. S4–9). The ^1^H NMR spectrum of **10** exhibited three methine proton signals (*δ*_H_ 6.65, 5.86, 4.17), two pairs of methylene groups (*δ*_H_ 2.69 and 1.88, 2.14 and 1.54), four methyl group singlets (*δ*_H_ 2.19, 1.93, 1.51 and 1.18), and an exchangeable proton (*δ*_H_ 7.28) was observed in DMSO‑*d*_6_. The ^13^C NMR spectrum exhibited fifteen carbon signals, including three carbonyl carbon signals (*δ*_C_ 196.7, 164.8, 159.0) and three quaternary carbon signals that appeared downfield from 70 ppm (*δ*_C_ 155.5, 152.9, and 71.4).

The interpretation of the 2D NMR spectral data allowed for the construction of two structural fragments. One fragment, isobutene motif, was established by interpreting the HMBC correlations from H-4’ (*δ*_H_ 1.93) to C-5’ (*δ*_C_ 19.1), C-2’ (*δ*_C_ 118.5), and C-1’ (*δ*_C_ 164.8), and H-2’ (*δ*_H_ 5.86) to C-4’ (*δ*_C_ 26.2) and C-5’ (*δ*_C_ 19.1) as well as the NOE correlation of H-2′ to H-4’ (Fig. S10). The other fragment was assigned to be tetrahydropyrrolo [1,2-c][1,3]oxazepine-1,5-dione. The ^1^H–^1^H COSY correlations corresponding to the connectivity of four carbons from C-5M to C-7 as well as the HMBC correlation of H-5 (*δ*_H_ 4.17) and C-8 (*δ*_C_ 71.4) allowed for the construction of the five-membered ring system. The HMBC correlations between H-5 to C-4 (*δ*_C_ 159.0) and H-8M (*δ*_H_ 1.51) and C-1 (*δ*_C_ 196.7) provided valuable information for the seven-membered ring moiety. Interpretation of the HMBC analysis allowed to identify correlations from H-2 (*δ*_H_ 6.65) and NH (*δ*_H_ 7.28) to C-3 (*δ*_C_ 152.9), NH(*δ*_H_ 7.28) to C-2’ (*δ*_C_ 118.5) and C-1 (*δ*_C_ 196.7), H-2 to C-1’ (*δ*_C_ 164.8) and C-3 (*δ*_C_ 152.9), indicating that the isobutene motif is positioned at C-3 of the oxazinone ring through the amide motif (Fig. S8b).

**Fig. 3:**
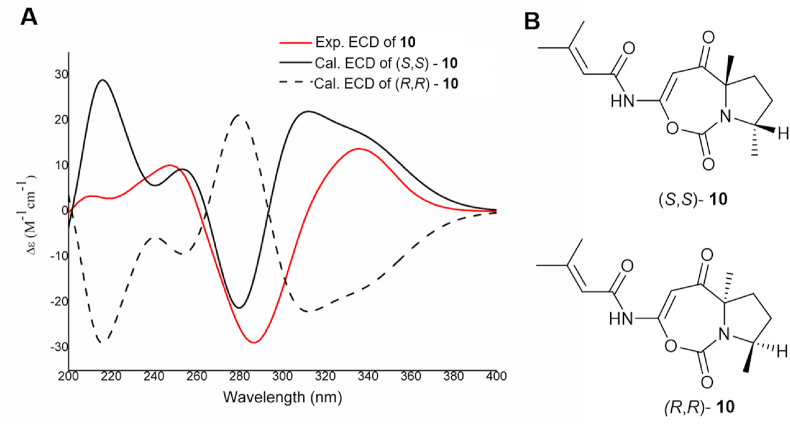
**A**. Measured and calculated ECD spectra for **10**. **B**. Two possible structures of **10** that contain *cis*-configuration between the methyl group at C8 and the hydrogen at C5.

In order to identify the relative configuration between the two methyl groups on C-5 and C-8, NOESY data was collected in which signals, albeit weak, showed a correlation between H-5 and H-8M (*δ*_H_ 1.51) (Fig. S11). This NOE correlation allowed us to assign the relative geometric relationship of H-5 and the methyl group at C-8 as a *cis* configuration. We utilized the empirical ECD to determine the absolute configuration of **10**. A comparison of the measured ECD curve with the predicted ECD spectra showed that the measured ECD of **10** matches with the calculated ECD curve of (5*S*,8*S*)-**10** (Fig. 3). Search in the natural product database, such as Antibase [27], only resulted in two analogues of **10** that contains the [5 + 7] heterocyclic carbamate ring system, which are cyclocarbamides, **12a** and **12b**, isolated from an unidentified *Steptoverticillium* sp [28]. and SB-315021 **13** from *Pseudomonas fluorescens* DSM 11579 [29] (Fig. 2).

Compound **11** was isolated as a yellow powder. Its molecular formula was determined to be C_14_H_20_N_2_O_2_ based on the HRESIMS data. The UV spectrum showed similar absorption to legonmycins at 250, 282 and 334 nm [3]. A comparison of its NMR spectra with those of known bohemamine analogues indicated that the ^1^H,^13^C and 2D NMR data of **11** were identical to NP25302 [3] (Figs. S12–15, Table S5).

Taken together, compound **10** is a new carbamate-containing [5 + 7] heterobicyclic alkaloid metabolite, which we named legoncarbamate, after its association with Legon, Ghana, the location of the University of Ghana. Compound **10** showed antibacterial activity against *E. coli* ATCC 25922 with a minimum inhibitory concentration (MIC) value of 3.1 μg/mL (Fig. S21).

Although various LC-MS-based dereplication tools, such as Global Natural Product Social molecular networking (GNPS) [18], have facilitated the targeted isolation of new SMs as well as rapidly dereplicating known ones, unambiguous identification of SMs still requires isolation and structure characterization. Recently, a new NMR-based machine learning tool, Small Molecule Accurate Recognition Technology (SMART 2.0) [26], has been developed for mixture analysis and subsequent discovery and characterization of SMs from environmental isolates. In our case, the minute amount of two semi-purified fractions from the culture of CT37 prevented further purification to obtain the pure substances for structural elucidation. Therefore, we applied this newly developed cheminformatic tool to assign the structures of the SMs present in these two fractions. Subsequently, the HSQC spectra of these two fractions were submitted to SMART NMR. SMART annotation combined with GNPS analysis and NMR interpretation suggested that the majority of the compounds in one fraction are two known bohemamine derivatives, bohemamine B **15** [24] and 5-chlorobohemamine C **16** [24] while bohemamine **17** [30] and bohemamine F **18** [25] in the other fraction (Figs. S16–S20, Tables S6–S7).

**Fig. 4:**
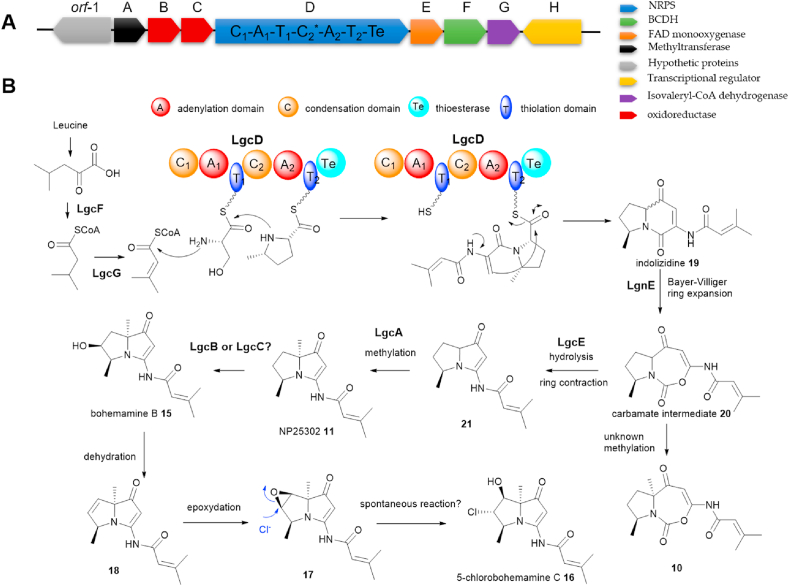
**A**. the organisation of the putative *lgc* BGC in *Streptomyces* sp. CT37. **B**. A proposed model of legoncarbamate **10** and bohemamine derivatives, **15**–**18**, identified in CT37.

The [5 + 7] heterobicyclic SMs containing a carbamate moiety are rare in the natural product inventory. Cyclocarbamide **12** [28] and SB315021 **13** [29] are the only two examples containing such a scaffold discovered thus far. It is worth to note that **13** was proposed to be the biosynthetic intermediate of brabantamide A **14**, an antibacterial SM isolated from plant-associated *Pseudomonas* strains [31,32]. Although brabantamide A **14** is a bicyclic pyrroloxazole SM, which is structurally different to all of PA SMs, its biosynthetic gene cluster is remarkably similar to the ones of PAs. One of the key differences between brabantamide A and PA SMs lies on the putative monooxygenase BraC which was proposed to catalyse a BV oxidative ring expansion to yield **13** as the biosynthetic intermediate, the co-occurring metabolite in the producing strains, followed by the ring re-arrangement via allylic 1,3-transposition [31]. It has yet remained to be determined whether **12** is originated from PA-like pathways or the **14**-like pathway. The proposed biotransformation catalysed by LgnC-like monooxygenases and the structural relationship among **10**, **12** and other co-occurring bohemamines suggested that they are likely to originate from the same PA biosynthetic pathway. Inspection of the structures of **10** and **12** led to the speculation that the precursors of the core of both compounds are (*S*)-5-methyl-proline and dehydroalanine, a dehydrated form of serine, a similar model to the one proposed in the recent report of bohemamines isolated from the environmental isolate, *Streptomyces* sp. CB02009 [33]. (*S*)-5-methyl-proline is a rare occurring motif in natural products and is mainly found in actinomycin complexes [34] and antibiotic agent A-54556H [35]. The bio-origin of this motif remains to be disclosed. *In silico* analysis of CT37 annotated draft genome led to the identification of a putative gene cluster (*lga*), spanning approximately 17.6 kbp. The centre of this cluster encodes one multidomain NRPS (LgaD, Fig. 4A). LgaD possesses a canonical arrangement of C_1_-A_1_-T_1_-C_2_-A_2_-T_2_-TE domains (Fig. 4A). The A domains of LgaD were predicted to activate serine and proline/5-methylproline, the same as the one in bohemamines [33], respectively. The gene product, LgaE, sharing high AA sequence identity (93%) with LgnC-like monooxygenases, is likely to catalyse the key biotransformation from indolizidine intermediate **18** to pyrrolizidine **20** via bicyclic carbamate intermediate **19** (Fig. 4B). Unlike the legonmycin pathway, the *lga* BGC contains a series of tailoring enzymes. For example, the gene product LgaA is likely to catalyse the methylation at C-7 position in bohemamine SMs. It is likely that a promiscuous methylation enzyme methylates the key intermediate, carbamate **19**, at C8 position during the catalytic cycle of LgaE, to generate legoncarbamate **10**. It remains to be elusive whether LgaA is responsible for the methylation at C8 position of **10**. The genes, *lga*F and *lga*G, encode branched-chain α-ketoacid dehydrogenase and isovaleryl CoA dehydrogenase, respectively, which are likely involved in catalysing the oxidative decarboxylation of 4-methyl-2-oxopentanoic acid to yield isovaleryl CoA and transfer isovaleryl CoA to 3-methyl pentenyl CoA (Fig. 4B, Table S8).

## 3 Material and method

### 3.1 General experimental procedures

The optical rotation was recorded using ADP 410 polarimeter (Bellingham + Stanley Ltd.2007, Kent, UK) equipped with a light emitting diode and interference filter. UV spectra were recorded on an Accela PDA detector (Waldbronn, Germany). IR spectra were obtained on a Fourier transform infrared (FTIR) spectrometer (2013, PerkinElmer, UK) equipped with an Attenuated Total Reflection (ATR, PerkinElmer, Buckinghamshire, UK) diamond cell for sample loading was used for infrared spectroscopy experiments. 1D and 2D NMR spectra were acquired on a Bruker AVANCE IIIHD400MHz (AscendTM14.1 T, UK) with Prodigy TCITM cryoprobe at 298 K in CD_3_OD and DMSO‑*d*_6_ (Goss Scientific, Massachusetts, MA, USA). Trimethylsilane (TMS) was used as an internal standard. HRESIMS data were obtained in positive ESI mode with a mass range of 100–2000 *m/z* (maximum resolution 30,000) on a Thermo Scientific MS system (LTQ XL/LTQ Orbitrap Discovery, Waldbronn, Germany). Reserpine (*m/z* 609.2807) was used as a lock mass for internal calibrant during data acquisition. The following instrument parameters were used: capillary voltage 45 V, spray voltage 4.5 kV, capillary temperature 200 °C, auxiliary gas flow rate 10–20 arbitrary units, and sheath gas flow rate 5 arbitrary units; furthermore, an automated full dependent MS-MS scan was applied. The injected samples were chromatographically separated in Thermo Instrument HPLC system (Accela PDA detector, Waldbronn, Germany), Accela PDA autosampler and Accela Pump (Agilent Technologies, Waldbronn, Germany) using a C18 (Sunfire 150 × 46 mm) column. The gradient elution for separation was CH_3_CN/H_2_O with 0.1% trifluoroacetic acid (TFA) (from 0% to 100% for 30 min, flow rate, 1.0 mL/min, UV detection max 340 nm).

### 3.2 Strain collection

Fifty-eight strains were used for genome scanning which was listed in Table S*2*. Among which 56 were provided by NCIMB while the remaining two were isolated from soil samples collected in Ghana as described in previous papers [23,36].

### 3.3 Cultivation and genomic DNA extraction

Strains were cultured in 50 mL ISP2 media and harvested after 7 days. Genomic DNA was extract using the following protocol to generate a library of genomic DNA. The genomic DNA in this study was extracted from 10 mL cell culture. Cell pellet was harvested by centrifugation and resuspended in 500 μL SET buffer. The cell suspension was mixed with lysozyme (4 mg/mL, final concentration) and incubated at 37 °C for 30 min. SDS (60 μL, 10% (w/v)) and NaCl (200 μL, 5 M) were then added to the mixture, followed by another incubation at 60 °C for 30 min. The protein was precipitated with the mixture of phenol, chloroform and isoamylol (500 μL, ratio of 25:24:1), and the resultant solution was mixed by vortex. The supernatant was separated by centrifuge and transferred to a new Eppendorf tube with isopropanol for DNA precipitation (0.8 vol). The precipitated DNA was washed with 75% (v/v) ethanol, followed by the second wash of 100% ethanol. DNA pellet was dried at room temperature and dissolved in sterile Milli-Q water (200 μL).

### 3.4 Genome scanning of strain library

MEME (Multiple EM for Motif Elicitation) was used for protein motif and degenerate primers design (Table S1) [37]. The DNA fragments were amplified from *Streptomyces* genomic DNA in the library using Taq DNA polymerase (Invitrogen, UK) with the degenerated primers.

The homologues genes of *lgn*C amplified from *Streptomyces* genomic DNA templates were inserted into pCR™ plasmid using TA Cloning™ Kit (Invitrogen, UK). The constructed plasmid was sequenced by the Dundee sequencing service.

### 3.5 Cultivation conditions

OSMAC strategy was applied [17] using 8 different fermentation broths (ISP2-ISP7, Modified Bennett's, Starch Casein, Table S1). These were selected based on the recommended medium for *Streptomyces* species, which differ with respect to carbon source and salt concentration [38].

The small scale culture (50 mL) of CT37 strain was prepared by inoculating a single colony of the bacteria in a solid medium of choice and incubated at 28 °C, 180 rpm for 7 days (Incu-shake FL16-2, SciQuip, Shrewsbury, UK). Subsequently, Diaion®HP-20 (3 g/50 mL, Mitsubishi Chemical Co., Binasco, Italy) was added to the fermentation cultures under sterile conditions. The flasks were left at the same shaking temperature and conditions for 18 h.

The culture broths were filtered under vacuum (Buchi pump V100, Buchi, Manchester, UK), and the HP-20 resin was rinsed with Milli-Q water and extracted exhaustively with methanol (MeOH, Fisher Chemical HPLC grade). All the methanol extracts were combined, and concentrated under reduced pressure (Buchi Rotavapor R200, Buchi, Manchester), and subjected to high-resolution electrospray ionization Liquid chromatography mass spectrometry analysis.

### 3.6 Large scale fermentation

For scale-up fermentation, a seed culture (50 mL) of CT37 was prepared following the same inoculation procedure as small scale cultivation. On the 3rd day, the seed culture was inoculated (1:100) into a 2 L Corning™ polycarbonate baffled flask (contains 250 mL broth). Each of the flasks (8 L in total) were plugged with Fisherbrand™ polyurethane foam stoppers (Fisher Scientific, UK). The cultures were fermented for 7 days under the same condition as described for the small scale fermentation.

### 3.7 HPLC-HRMS/MS analysis and metabolites annotation using GNPS and SMART 2.0

The MS/MS data were converted from. RAW to. mzXML files using the ProteoWizard MSconvert software [39]. A molecular network was generated using Feature-Based Molecular Networking (FBMN) workflow [40] on Global Natural Product Social networking (GNPS) [18] (https://gnps.ucsd.edu). The mass spectrometry data were pre-processed with MZmine v2.38 [41] and exported to GNPS for FBMN analysis. The data were filtered to remove all MS/MS fragment ions within ±17 Da of the precursor *m/z*. MS/MS spectra were window filtered by choosing only the top 6 fragment ions in the ±50 Da window throughout the spectrum. The precursor ion mass tolerance was set to 0.02 Da with an MS/MS fragment ion tolerance of 0.02 Da to create consensus spectra. The consensus spectra that contained fewer than four spectra were discarded. The edges were filtered to ensure a cosine score above 0.65 and more than four matched peaks. The edges between two nodes were kept in the network if each of the nodes appeared in each other's respective top 15 most similar nodes. The spectra in the network were then searched against GNPS spectral libraries [18] and annotated by the DEREPLICATOR [42]. The library spectra were filtered in the same manner as the input data, where a score above 0.65 and at least 4 matched peaks are required. The created molecular network was visualized using Cytoscape software v3.4.0 (Seattle, WA, US) [43].

Smart 2.0 was used for rapid structure prediction of major constituents from crude extracts and fractions (https://smart.ucsd.edu/classic). The experimental HSQC data was annotated to generate a digitalized HSQC spectrum for library alignment and analysis. Top 6 structures were listed based on cosine similarity score [19].

### 3.8 HPLC isolation

The compounds of interest were identified in S2 fraction, hence further fractionation was carried out in this fraction using High Pressure Liquid Chromatography (HPLC, Agilent Technologies 1260 infinity, Waldbronn, Germany). The purification was performed using a linear gradient from 10% H_2_O:MeOH (95:5) to 100% MeOH for 30 min with a solvent flow rate of 1.5 mL/min (C-18 ACE 10 μM 10 × 250 mm column). As a result, **10** (1.0 mg), **11** (1.5 mg) were isolated.

Legoncarbamate (**10**): white powder, ${\left[\alpha \right]}_{\text{D}}^{25}$+11.3 (c 0.50, MeOH); UV(MeOH) *λ*_max_ (log *ε*) 275 nm; ECD (c = 1.0 mg/mL, MeOH) *λ*_max_ (Δε) 338(+13), 284(-30), 248(+10), 215(-5) nm; IR νmax 3404,1742, 1728, 1671, 1615 cm^−1^; ^1^H and ^13^C NMR, Table S4; HRESIMS *m/z* 293.1502 [M + H]^+^.

NP25302 (**11**): yellow powder, ${\left[\alpha \right]}_{\text{D}}^{25}$ +9.2 (c 0.50, MeOH); UV(MeOH) *λ*_max_ (log *ε*) 250, 282, 334 nm; ECD (c = 1.0 mg/mL, MeOH) *λ*_max_ (Δε) 338(+12), 284(-25), 248(+6), 212(-4) nm; IR *ν*_max_ 3283, 3180, 1711, 1642, 1571 and 1497 cm^−1^; ^1^H and ^13^C NMR, Table S5; HRESIMS *m/z* 249.1603 [M + H]^+^.

### 3.9 Antibacterial assay

Minimum inhibitory concentrations (MIC) were determined following the antibacterial assay protocols described in accordance with standards recommended by the National Committee for Clinical Laboratory Standards (NCCLS) [44].

A panel of pathogens was tested against including *Escherichia coli* (ATCC 25922), *Pseudomonas aeruginosa* (ATCC 27853), *Streptococcus B* (ATCC 12386), *Staphylococcus aureus* ATCC 25923, *Staphylococcus haemolyticus* clinical isolate 8‐7A, *Staphylococcus epidermidis* ATCC 35984 and *Enterococcus faecalis* ATCC 29212. All bacteria were cultured in Mueller–Hinton broth. The assays were performed in serial dilutions (in triplicates) in 96-well plates (Nunc, Thermo Fisher Scientific, UK), wherein a 50 μL suspension (log phase) of bacteria was incubated overnight at 37 °C and then supplemented with 50 μL of the test compound or fraction. The positive control consisted of the bacteria plus Milli-Q water (no test compound), while the negative control comprised the growth media and Milli-Q water. The absorbance was recorded after 24 h (OD_600_) in a SpectraMax ABS Plus (Molecular Device) plate reader. The MIC was defined as the lowest concentration of a drug that inhibited visible bacterial growth.

### 3.10 Measured and calculated electronic circular dichroism (ECD)

ECD spectra were measured on a Jasco J810 Spectropolorimeter. Cell path length 0.05 cm. Data pitch 0.2 nm, Scanning range 400-200 nm at a rate of 20 nm/min with response 1.0 s, bandwidth 1 and three accumulations.

In general, conformational analyses were carried out via random searching in the Sybyl-X 2.0 using the MMFF94S force field with an energy cutoff of 2.5 kcal/mol (Sybyl Software, version X 2.0; Tripos Associates Inc.: St. Louis, MO, 2013). The results showed eight lowest energy conformers for the compound. Subsequently, the conformers were re-optimized using DFT at the PBE0-D3(BJ)/def2-SVP level in MeOH using the polarizable conductor calculation model (SMD) by the GAUSSIAN 09 program (Gaussian, Inc., Wallingford CT, 2009). The energies, oscillator strengths, and rotational strengths (velocity) of the first 30 electronic excitations were calculated using the TDDFT methodology at the CAM-B3LYP-D3(BJ)/def2-TZVP level in MeOH. The ECD spectra were simulated by the overlapping Gaussian function (half the bandwidth at 1/e peak height, sigma = 0.30 for all) [45]. To get the final spectra, the simulated spectra of the conformers were averaged according to the Boltzmann distribution theory and their relative Gibbs free energy (ΔG). By comparing the experiment spectra with the calculated model molecules, the absolute configuration of the chiral centres was determined to be 5*S*, 8*S*.

## 4 Conclusion

In conclusion, we applied genomic scanning strategy to probe our culture collection to identify the potential producers of pyrrolizidine alkaloids (PAs). Fifteen out of fifty-eight strains tested have the capacity of producing PAs. One of these thirteen potential producers was found to produce known bohemamine derivatives and a new [5 + 7] heterobicyclic alkaloid, legoncarbamate **10**, using a combination of chemical workup, NMR-based structural elucidation, SMART 2.0 technology in conjunction with LC-MS-based GNPS analysis. The absolute stereochemistry of legoncarbamate **10** was determined by a comparison of measured and calculated ECD spectra. Legoncarbamate **10** displays antibacterial activity against Gram-negative *E*. *coli* ATCC 25922 with an MIC value of 3.1 μg/mL. Finally, a biosynthetic model of legoncarbamate and other known bohemamine derivatives was proposed based on bioinformation analysis and current knowledge of PA biosynthesis.

## Author contributon

Q.F., H.D. Conceptualization; Q.F., H.D. Data curation; Q.F., L.W., C.U., S.W. M.M., H.D. Formal analysis; H.D., K.K., S.L., C.P., Funding acquisition; Q.F., L.W., C.U., S.W. M.M., H.D. Investigation; Q.F., H.D. Methodology; H.D. Project administration; K.K. and H.D., Resources; Q.F., L.W., C.U., S.W. Software; H.D. Supervision; Q.F., L.W., C.U., S.W. M.M., Validation; Q.F., L.W., C.U., S.W. M.M., Visualization; Q.F., H.D. Roles/Writing – original draft; K.K. and H.D., Writing – review & editing.

## Declaration of competing interest

All of the authors declare no conflict of interest.
