# Supplementary material for: Genomic scanning enabling discovery of a new antibacterial bicyclic carbamate-containing alkaloid
Source: Synth Syst Biotechnol. 2021 Jan 20;6(1):12–9. doi: 10.1016/j.synbio.2021.01.002 (PMC7820566; doi:10.1016/j.synbio.2021.01.002)
Supplement: Multimedia component 1 [file mmc1.docx]

**Supporting Information**

**Contents:**

Figure S1. MEME protein motif

Table S1. Primer list used in this study

Table S2. Strains list used in this study

Table S3. Culture media used in the study

Figure S2. Amino acid alignment of LgnC and amino acid fragments of LgnC-like monooxygenase obtained from our genomic scanning.

Figure S3. GNPS network of CT37 crude extract with the highlight of PA metabolites.

Figure S4. HRLC-MS of compound **10**

Figure S5. MS^2^ fragmentation pattern of compound **10**

Figure S6 ^1^H-NMR of compound **10**

Figure S7. HSQC of compound **10**

Figure S8. HMBC of compound **10**

Figure S9. COSY of compound **10**

Figure S10-11. NOESY of compound **10**

Table S4. Comparison of the ^1^H and ^13^C -NMR of **10** with the NMR data of **12a** reported in literatures

Figure S12. ^1^H-NMR of compound **11**

Figure S13. HSQC of compound **11**

Figure S14. HMBC of compound **11**

Figure S15. COSY of compound **11**

Table S5. Comparison of the ^1^H and ^13^C -NMR of **11** with the NMR data reported in literatures

Figure S16. Smart 2.0 and HRLC-MS dereplication of compound **15**

Figure S17. Smart 2.0 and HRLC-MS dereplication of compound **16**

Figure S18. Isotope pattern of compound **16**

Table S6. Comparison of the ^1^H and ^13^C -NMR of **15 and 16**with the NMR data reported in literatures

Figure S19. Smart 2.0 and HRLC-MS dereplication of compound **17**

Figure S20. Smart 2.0 and HRLC-MS dereplication of compound **18**

Table S7. Comparison of the ^1^H and ^13^C-NMR of **17 and 18** with the NMR data reported in literatures

Table S8. MIC value of tested compounds against a panel of pathogens

Figure S21. MIC curve of compound **10**

Table S9. Deduced functions of ORFs in *lga* biosynthetic gene cluster


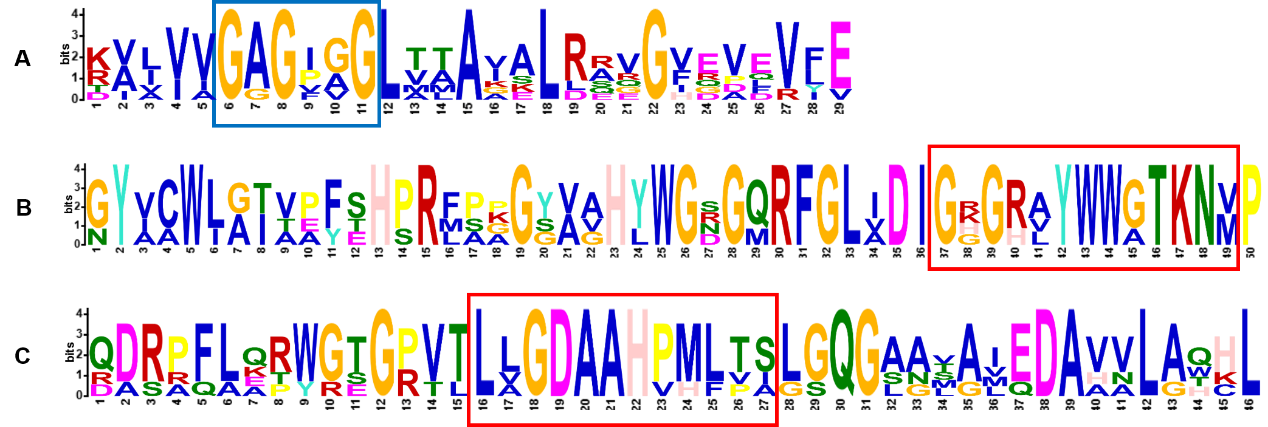


Figure S1A. MEME protein motif elicitor analysis indicated that three key motifs are highly conserved among LgnC-like monooxygenases in the identified PA biosynthetic pathways compared with other known Bayer Villiger monooxygenases that catalyse various chemical reactions. **A**. the conserved FAD-binding motifs among other bacterial BV monooxygenases identified from natural product biosynthetic pathways. **B** and **C**. two conserved motifs that are only present in LgnC-like BV monooxygenases. The amino acid sequences in the red boxes were used to design degenerate primers for PCR-screening (forward primer in **B** and reverse primer in **C** (Table S1)). The amino acid sequences used for this alignment are LgnC (accession no: AIZ66878.1) in the biosynthesis of legonmycins [1] ( LpiC (accession no: AFV70302.1) in the biosynthesis of lipocarbamates [2] PxaB (accession no: ALJ96774.1) in the biosynthesis of pyrrolizixenamide [3] AzeC (accession no: NP_252018.1) in the biosynthesis of azetidomononamide [4] BhmK (accession no: OKJ62000.1) in the biosynthesis of bohemamines [5] ForX (AQP25572.1) in the biosynthesis of Formicamycin [6] MtmOIV (3FMW_A) in the biosynthesis of Mithramycin [7] CalD (BAP05592.1) in the biosynthesis of calyculin [8].


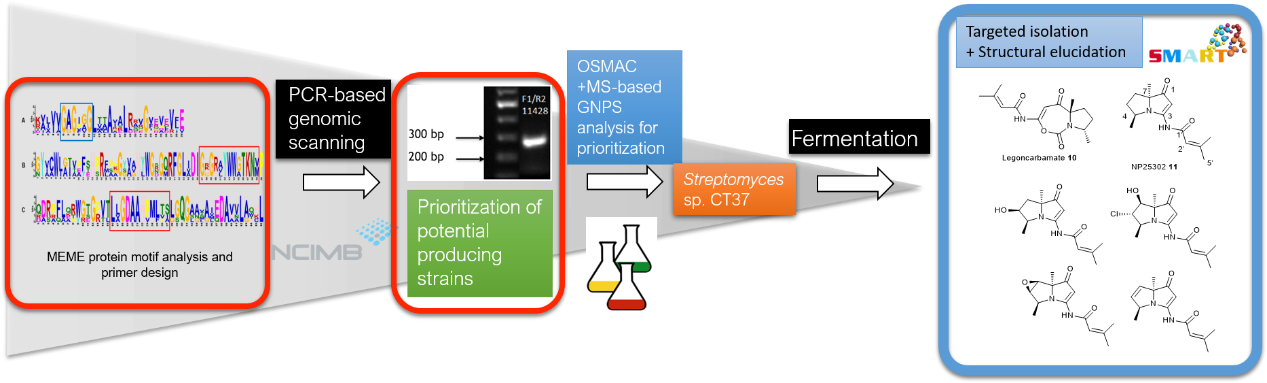


Figure S1B. a workflow scheme of PCR-based genomic scanning to prioritize the potential producing strains, followed by identification of a producing strain in the lab conditions using various microbiological and MS-based analytical methods. After large-scale fermentation, targeted isolation will afford new compounds for structural elucidation and bioactivity evaluations.

| **Table S1**. Primers used in this study | | |
| --- | --- | --- |
| **Primer** | **Sequence (5’-3’)** | **Function** |
| Deg_C_F | GGNGCNGAYGGNTTYCAYWSNGCNGTNMG | Amplification of *LgnC* homologues |
| Deg_C_R | RCATNGGRTGNGCNGCRTCNCCNARNARNGT | Amplification of *LgnC* homologues |

| **Table S2.** Strains used in this study | |
| --- | --- |
| **NCIMB Number** | **Taxon name** |
| NCIMB 8229 | *Streptomyces rimosus subsp. rimosus* |
| NCIMB 8591 | *Streptomyces* sp. TresnerAE-432 |
| NCIMB 8851 | *Streptomyces cinnamoneus* |
| NCIMB 8936 | *Streptomyces distallicus* |
| NCIMB 9000 | *Streptomyces lavendulae* |
| NCIMB 9343 | *Streptomyces kanamyceticus* |
| NCIMB 9444 | *Streptomyces californicus* |
| NCIMB 9598 | *Streptomyces echinatus* |
| NCIMB 9603 | *Streptomyces graminofaciens* |
| NCIMB 9606 | *Streptomyces griseolus* |
| NCIMB 9615 | *Streptomyces coeruleorubidus* |
| NCIMB 9618 | *Streptomyces globisporus subsp. globisporus* |
| NCIMB 9619 | *Streptomyces glaucescens* |
| NCIMB 9620 | *Streptomyces coeruleorubidus* |
| NCIMB 9621 | *Streptomyces* sp. Gause5051/56 |
| NCIMB 9796 | *Streptomyces globisporus subsp. globisporus* |
| NCIMB 9802 | *Streptomyces coralus* |
| NCIMB 9806 | *Streptomyces citreofluorescens* |
| NCIMB 9811 | *Streptomyces griseoplanus* |
| NCIMB 9819 | *Streptomyces endus* |
| NCIMB 9821 | *Streptomyces griseofuscus* |
| NCIMB 9825 | *Streptomyces griseoincarnatus* |
| NCIMB 9828 | *Streptomyces goshikiensis* |
| NCIMB 9831 | *Streptomyces cyanoalbus* |
| NCIMB 9845 | *Streptomyces griseomycini* |
| NCIMB 9846 | *Streptomyces griseorubens* |
| NCIMB 9853 | *Streptomyces griseoviridis* |
| NCIMB 9857 | *Streptomyces xantholiticus* |
| NCIMB 9887 | *Pseudomonas putida* |
| NCIMB 9979 | *Streptomyces corchorusii* |
| NCIMB 10029 | *Streptomyces griseus subsp. erizensis* |
| NCIMB 10030 | *Streptomyces cremeus* |
| NCIMB 10042 | *Streptomyces coelescens* |
| NCIMB 10210 | *Streptomyces diastatochromogenes subsp. luteus* |
| NCIMB 10490 | *Streptomyces griseoflavus* |
| NCIMB 10505 | *Streptomyces fulvissimus* |
| NCIMB 10721 | *Streptomyces fragmentans* |
| NCIMB 10722 | *Streptomyces fragmentans subsp. aquatica* |
| NCIMB 10969 | *Streptomyces mediolani* |
| NCIMB 10975 | *Streptomyces albofaciens* |
| NCIMB 10989 | *Streptomyces distallicus* |
| NCIMB 11004 | *Streptomyces fumigatiscleroticus* |
| NCIMB 11008 | *Streptomyces flaviscleroticus* |
| NCIMB 11081 | *Streptomyces gedanensis* |
| NCIMB 11904 | *Streptomyces* sp. Lindenbein1952 Tu365 |
| NCIMB 11968 | *Streptomyces cattelaya* |
| NCIMB 12599 | *Streptomyces exfoliatus* |
| NCIMB 12677 | *Streptomyces glaucus* |
| NCIMB 12680 | *Streptomyces fungicidicus* |
| NCIMB 12681 | *Streptomyces cinnamoneus subsp. forma azacoluta* |
| NCIMB 12785 | *Streptomyces clavuligerus* |
| NCIMB 12828 | *Rhodococcus equi* |
| NCIMB 12830 | *Streptomyces puniceus* |
| NCIMB 12831 | *Streptomyces echinoruber* |
| NCIMB 12860 | *Streptomyces crystallinus* |
| NCIMB 13271 | *Actinosynnema mirum* |
| Soil isolate | *Streptomyces* sp. CT37 |
| Soil isolate | *Streptomyces* sp. RK44 |

| **Table S3.** Culture media used in the study | |
| --- | --- |
| **Media** | **Composition** |
| Modified Bennett's (MB) | Glycerol 10g, Bacto-Casitone 2g, Yeast Extract 1g, Lab-Lemco 0.8g |
| ISP2 | Glucose 4 g, Yeast extract 4 g, Malt extract 10 g, milliQ water 1L |
| ISP3 | pH = 7.2, Oatmeal 20 g, milliQ water 1L Trace elements: 1 mL (FeSO_4_.7H_2_O 0.1g, MnCl_2_.4H_2_O 0.1g, ZnSO_4_.7H2O, 0.1g per liter) |
| ISP4 | pH 7.2 Solution 1: 500 mL, Difco soluble starch 10.0 g/500 mL water Solution 2: 500 mL, K_2_HPO_4_ 1 g, MgSO_4_ 7H_2_O 1g, NaCl 1g, (NH_4_)_2_SO_4_ 1g, CaCO_3_ 1g, Trace elements (above) 1 mL, milliQ water 1L |
| ISP5 | pH = 7.2 L-asparagine (anhydrous) 1.0g, glycerol 10.0 g, K_2_HPO_4_ 1.0 g (anhydrous), trace salts solution (above) 1.0 mL, milliQ water 1L |
| ISP6 | Bacto-peptone 15 g, proteose peptone (Difco) 5 g, ferric ammonium citrate 0.5 g, dipotassium phosphate 1 g, sodium thiosulfate 0.08 g, Bacto-Yeast extract (Difco) 1 g, milliQ water 1L |
| ISP7 | pH=7.2  Glycerol 15g, L-tyrosine 0.5 g, L- asparagine (Difco) 1 g, K2HPO4 (anhydrous) 0.5g, MgSO_4_۰7H₂O, 0.5g NaCl, 0.5g FeSO_4_.7H_2_O, 0.01g Trace salts solution (above) 1 mL, milliQ water 1L |
| Starch Casein (SC) | Starch 10g, casein 0.3g, KNO₃ 2g, NaCl 2g, K₂HPO₄ 2g, MgSO₄۰7H₂O 5mg, CaCO₃ 2mg, FeSO₄∙7H₂O 10mg |


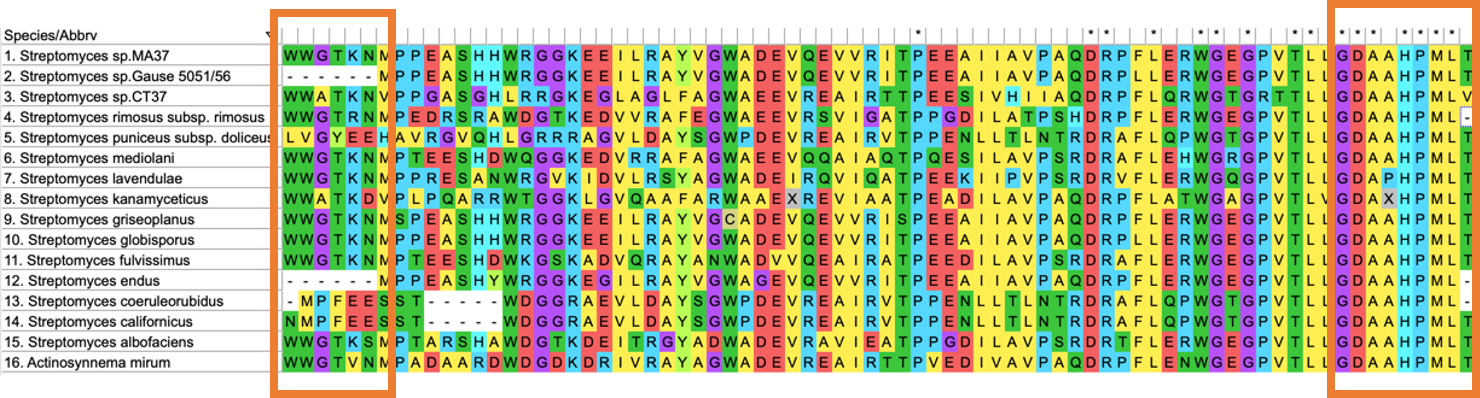


Figure S2. Amino acid alignment of internal AA fragments of LgnC and LgnC-like monooxygenase obtained from our genomic scanning. Both forward and reverse degenerate primers were indicated in the red boxes. The obtained DNA sequences were translated into amino acid sequences using FramePlot. For entries 2 and 12, the quality of DNA sequences at the beginning was low, resulting in no translation of amino acid sequences.

Figure S3. GNPS network of CT37 crude extract with the highlight of PA metabolites.


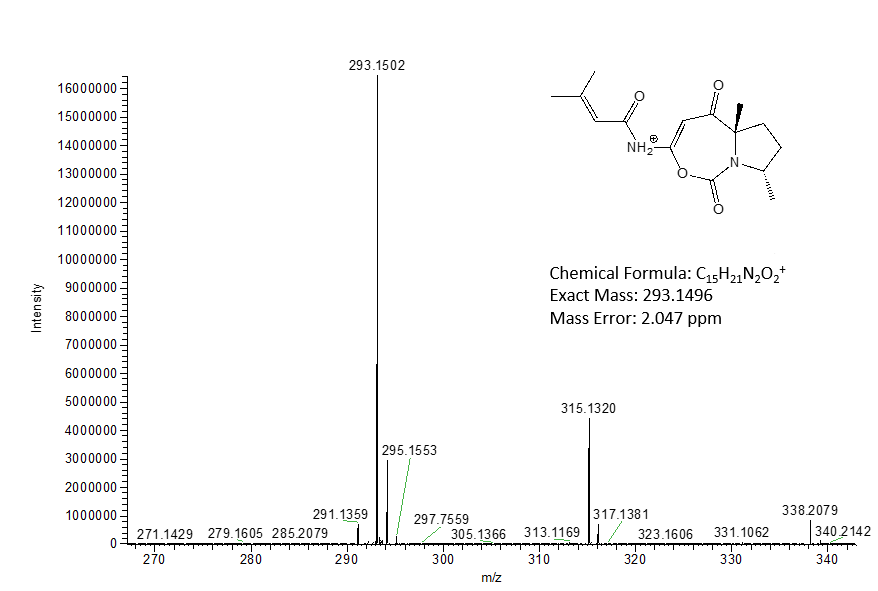


Figure S4. HR-LCMS of compound **10**


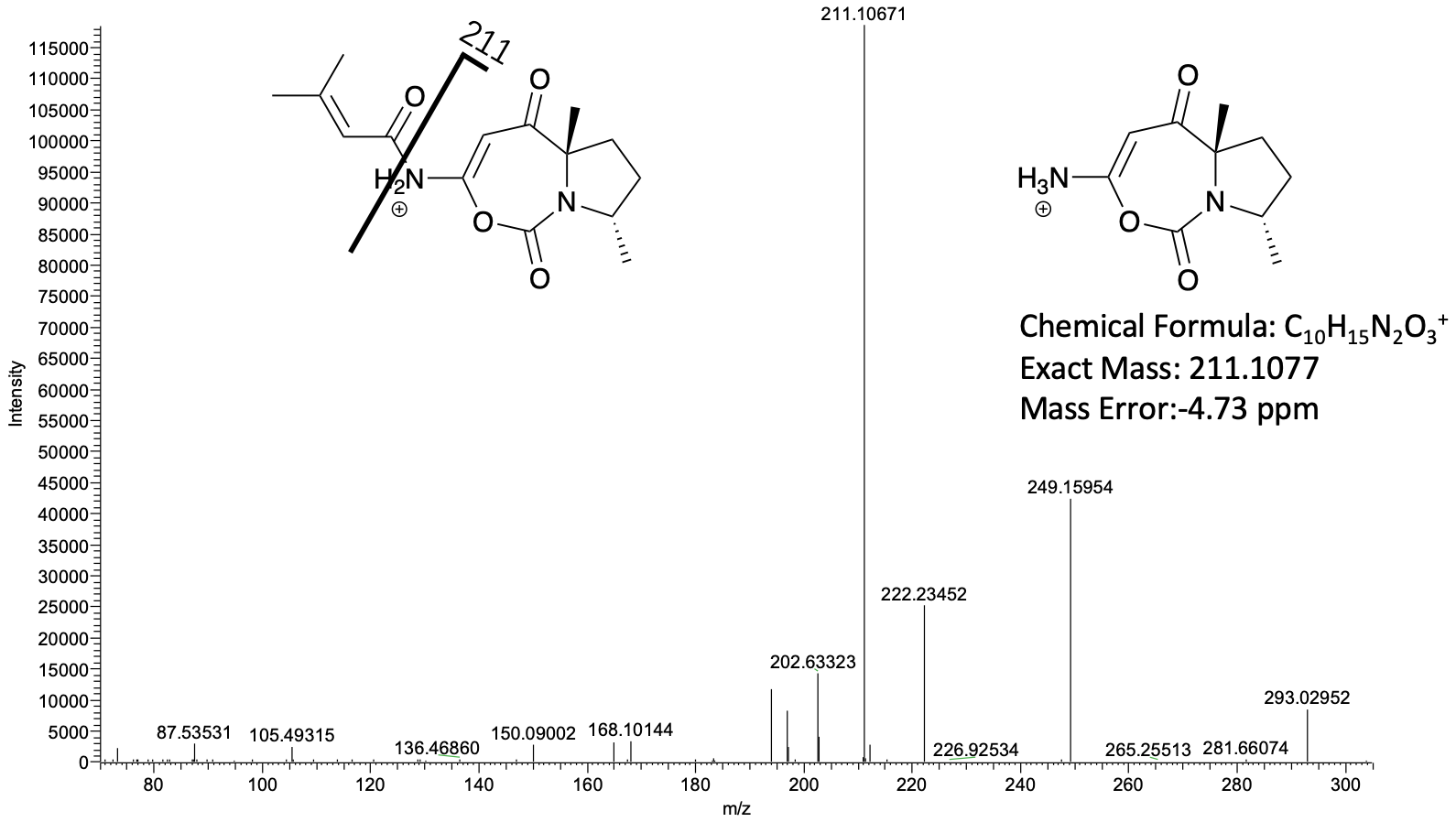


Figure S5. MS^2^ fragmentation pattern of compound **10**

H-7b

H-6b

H-6a

Solvent

H-8M

H-4’

H-5M

H-5’

H-7a

H-5

H-2

H-2’

Figure S6a. ^1^H-NMR of compound **10** in CD_3_OD (400MHz, 298K)

Solvent

Solvent

H-7a

NH

H-2

H-2’

H-5

H-4’

H-5’

H-6a

H-6b

H-7b

H-8M

H-5M

Figure S6b. ^1^H-NMR of compound **10** in DMSO (400MHz, 298K)

Figure S7. HSQC of compound **10** in CD_3_OD (400MHz, 298K)

Figure S8a. HMBC of compound **10** in CD_3_OD (400MHz, 298K)

Figure S8b.HMBC of compound **10** in DMSO (400MHz, 298K)

H-8M

H-5’

H-5M

H-2’

H-5

H-7a

H-6b

H-6a

H-4’

H-7b

Solvent

H-8M

H-5’

H-5M

H-2’

H-5

H-7a

H-6b

H-6a

H-4’

H-7b

Solvent


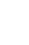

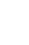


H_2_O

H-2

H-2’

Figure S9. COSY of compound **10** in CD_3_OD (400MHz, 298K)


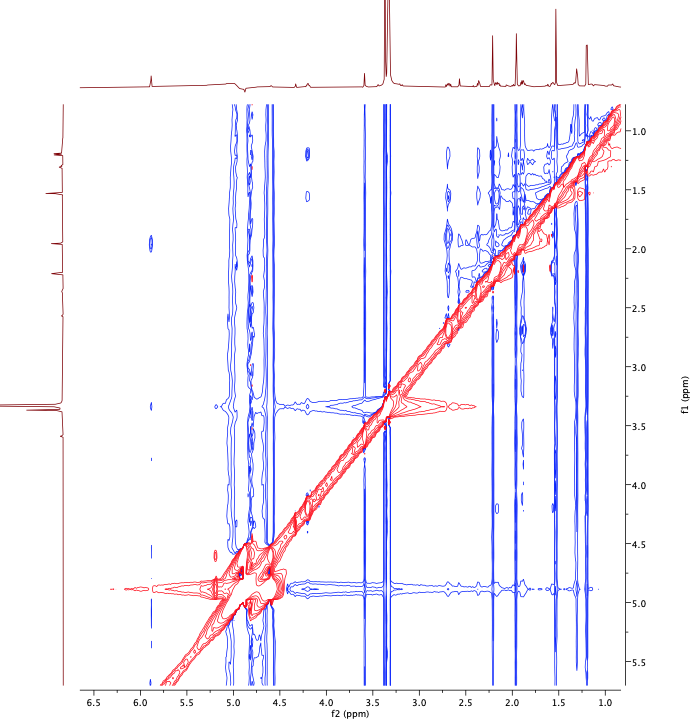

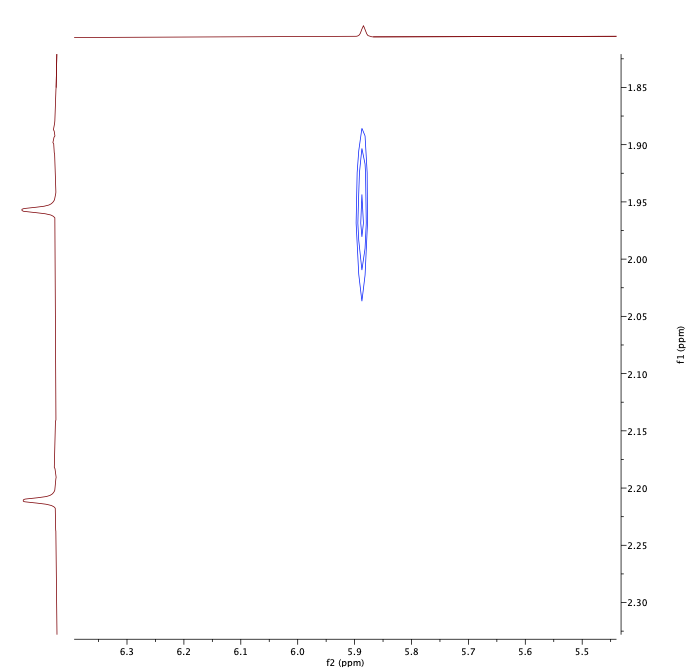

H-5’

H-4’

H-2’

H-8M

H-5’

H-5M

H-7a

H-6b

H-6a

H-4’

H-7b

Solvent

H-8M

H-5’

H-5M

H-5

H-7a

H-6b

H-6a

H-4’

H-7b

Solvent

H-2’

Figure S10. NOESY of legoncarbamate **10** in CD_3_OD (400MHz, 298K) shows correlation between H-2’ and H-4’.


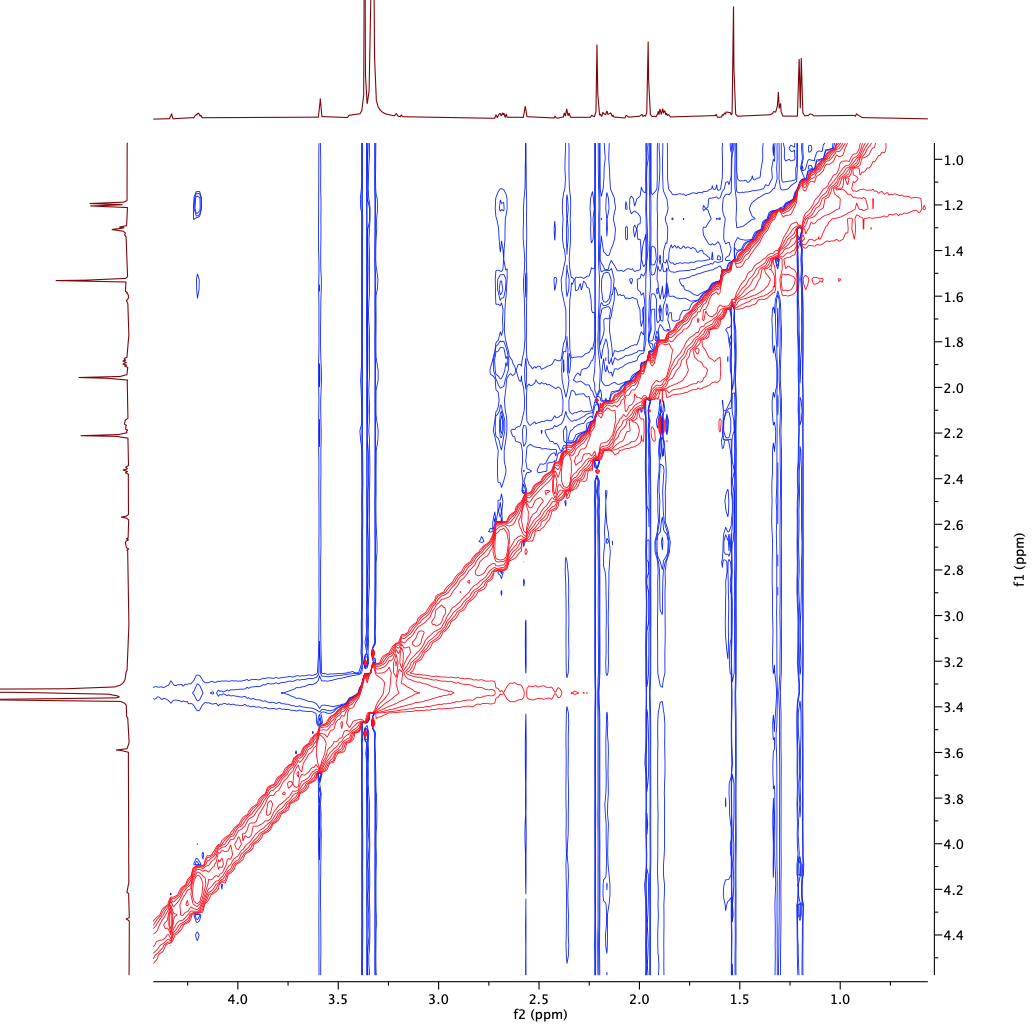


H-8M

H-5’

H-5M

H-5

H-7a

H-6b

H-6a

H-4’

H-7b

Solvent

H-8M

H-5’

H-5M

H-5

H-7a

H-6b

H-6a

H-4’

H-7b

Solvent

H-5

H-8M

H-5M

Figure S11. NOESY of legoncarbamate **10** in CD_3_OD (400MHz, 298K) shows *cis* correlation between H-8M and H-5

| Position | legoncarbamate 10 | | cyclocarbamide A 12a | |
| --- | --- | --- | --- | --- |
|  | *δ*_C_/ppm, type | *δ*_H_/ppm, mult. (J/Hz) | *δ*_C_/ppm, type | *δ*_H_/ppm, mult. (J/Hz) |
| 5’ | 19.1 | 2.19(3H,s) | 22.4 | 1.00,d |
| 4’ | 26.2 | 1.93(3H,s) | 22.4 | 1.01,d |
| 3’ | 155.5 | - | 25.9 | 2.18 m |
| 2’ | 118.5 | 5.86(1H,s) | 45.9 | 2.28,d |
| 1’ | 164.8 | - | 169.7 | - |
| 3 | 152.9 | - | 150.0 | - |
| 2 | 95.8 | 6.65(1H,s) | 110.3 | - |
| 2-M | - | - | 13.3 | 1.73.s |
| 1 | 196.7 | - | 189.5 | - |
| 8 | 71.4 | - | 63.2 | 4.47(dd) |
| 8-Me | 21.4 | 1.51(3H,s) | - | - |
| 7 | 35.0 | 2.69,1.88(2H,m) | 24.9 | 2.75,1.97 |
| 6 | 29.7 | 2.14,1.54(2H,m) | 23.8 | 1.97,1.86 |
| 5 | 59.3 | 4.17(1H,m) | 47.7 | 3.45-3.55 |
| 5-Me | 17.7 | 1.18(3H,d) | - | - |
| 4 | 159.0 | - | 150.0 | - |
| NH | - | 7.28,s | - | 7.61,s |

Table S4. Comparison of the ^1^H and ^13^C -NMR of **10** (DMSO, ^1^H-NMR at 400 MHz, ^13^C-NMR at 100 MHz) with the NMR data of **12a** (CDCl_3_, ^1^H-NMR at 400 MHz, ^13^C-NMR at 25 MHz) reported in the literature.

H-5a

H-5b

H-6b

H-6a

H-7M

H-4’

H-5’

H_2_O

Solvent

H-2’

H-2

H-4

H-4M

Figure S12. ^1^H-NMR of compound **11** in CD_3_OD (400MHz, 298K)

H-4’

H-7M

H-5’


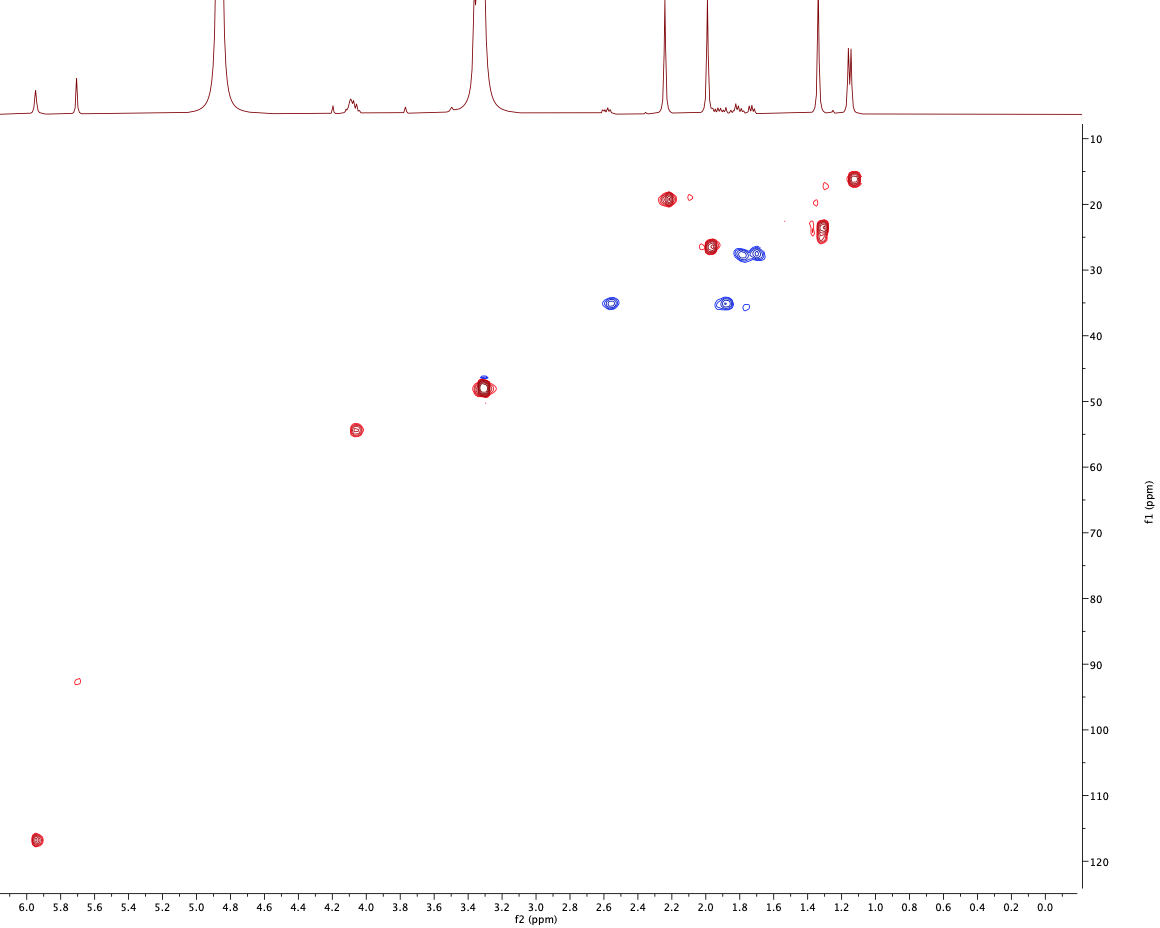


H-6b

H-6a

H-5b

H-5a

H-2’

H-2

H-4

Solvent

H-4M

Figure S13. HSQC of compound **11** in CD_3_OD (400MHz, 298K)


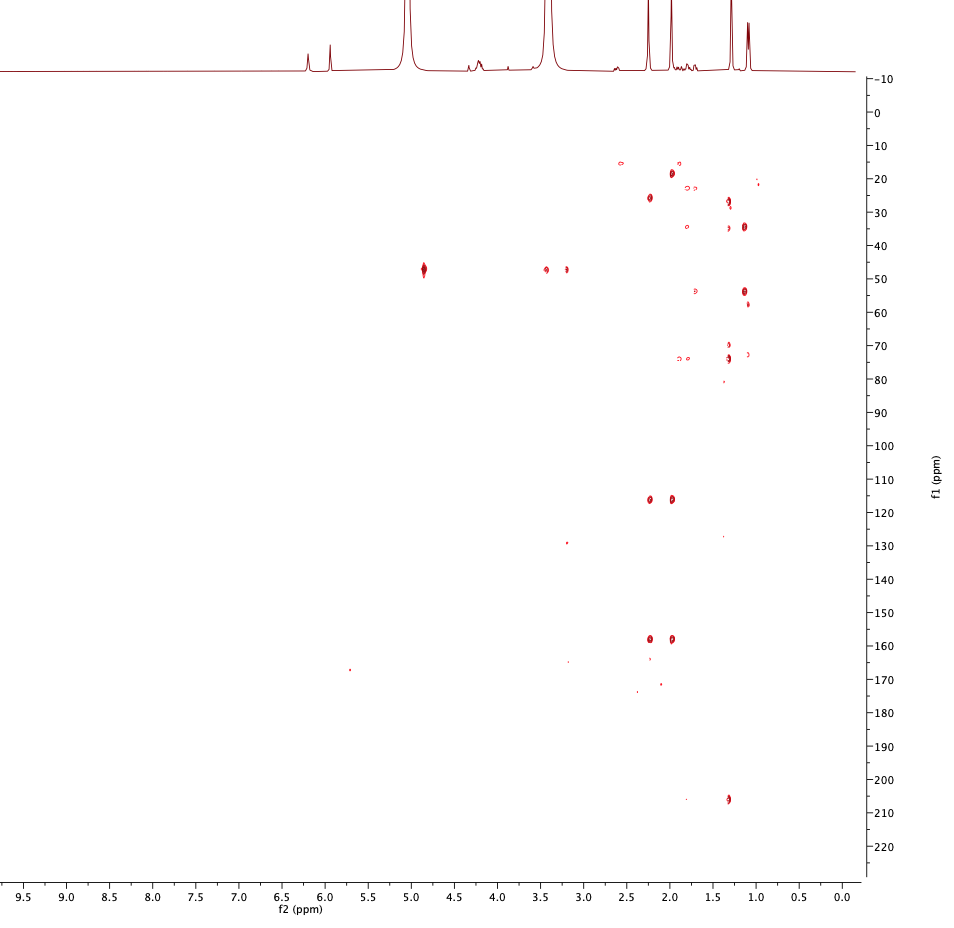


H-6a

H-6b

H-5a

H-5b

H-4’

H-5’

H-4M

H-7M

Solvent

H-4

H-2

H-2’

Figure S14. HMBC of compound **11** in CD_3_OD (400MHz, 298K)


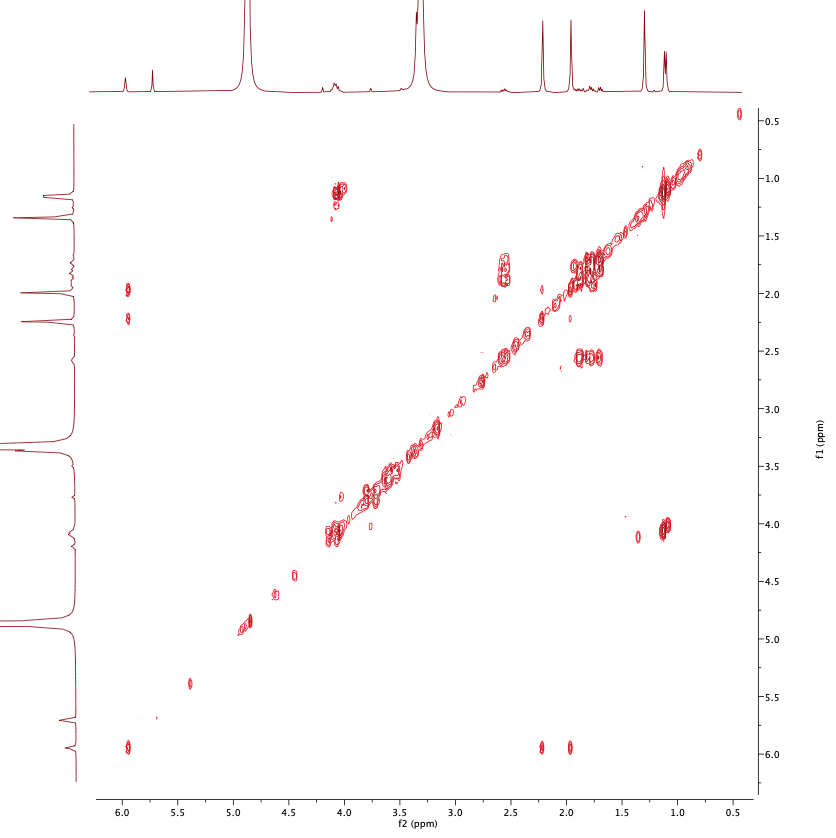


H-4

H-4’

H-5’

H-7M

H-4M

H-5’

H-4M

H-2’

H-2

H-4

H-5a

Solvent

H-7M

H-5b

H-6a

H-6b

H_2_O

H-4’

H-2’

H-2

H-5a

Solvent

H-5b

H-6a

H-6b

H_2_O

H-5’

Figure S15. COSY of compound **11** in CD_3_OD (400MHz, 298K)

| No | NP25302  ^13^C ^1^H, mult. | | 11  ^13^C ^1^H, mult. | |
| --- | --- | --- | --- | --- |
| 1 | 205.7 | - | 206.7 | - |
| 2 | 94 | 5.75(1H,s) | 92.8 | 5.70(1-H,s) |
| 3 | 167.7 | - | 168.3 |  |
| 4 | 55.2 | 4.08(1H,m) | 54.4 | 4.06(1H,dq) |
| 5 | 35.8 | 2.46(1H,m),1.83(1H,m) | 35.2 | 2.55(1H,ddd),1.87 (1H,d) |
| 6 | 28.7 | 1.85(1H,m),1.69(1H,m) | 27.7 | 1.81(1H,m),1.71(1H,m) |
| 7 | 75.1 |  | 73.2 |  |
| 4-Me | 17.8 | 1.18(3H,d,6.5) | 16.1 | 1.12(3H,d) |
| 7-Me | 25 | 1.34(3H,s) | 23.5 | 1.30(3H,s) |
| 1’ | 164.6 |  | 164.4 |  |
| 2’ | 118.1 | 6.01(1H,s) | 116.6 | 5.94(1H,s) |
| 3’ | 158.6 |  | 158.7 |  |
| 4’ | 28.1 | 1.93(3H,s) | 26.4 | 1.97(3H,s) |
| 5’ | 20.9 | 2.23(3H,s) | 19.2 | 2.22(3H,s) |

Table S5. Comparison of the ^1^H and ^13^C-NMR of **11** with the NMR data reported in literature for NP25302 [9] (CD_3_OD, ^1^H-NMR at 400 MHz, ^13^C-NMR at 100 MHz)

A

B


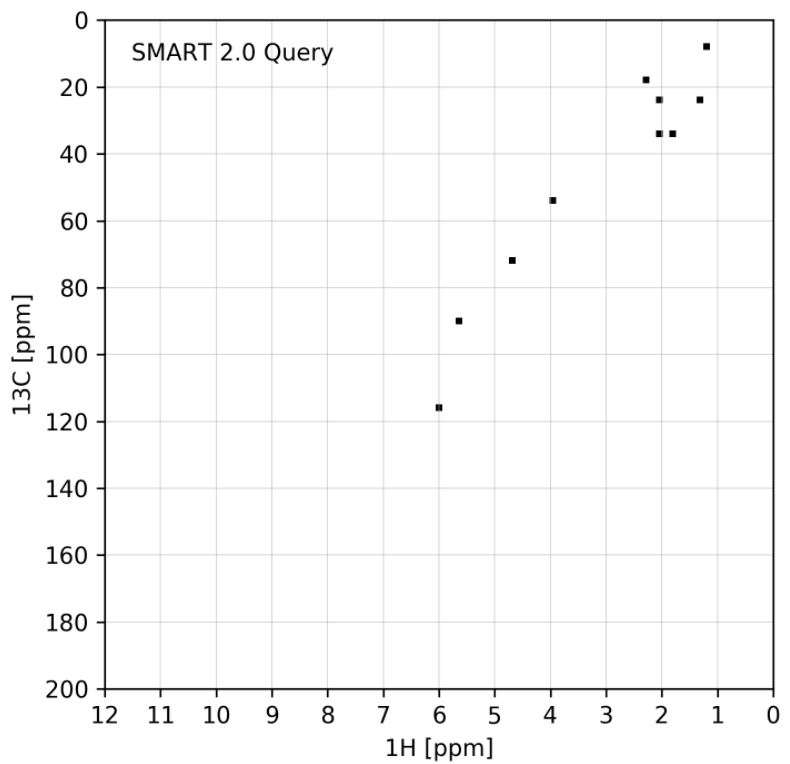

D

C

Figure S16. Smart 2.0 and HR-LCMS help with dereplication of **15.** (A) HSQC NMR of semi pure **15** in CD_3_OD (400MHz, 298K). (B) Digitalized HSQC of semi pure **15**. (C) SMART 2.0 results (top 6 structures based on cosine similarity score) suggests the structure as bohemamine B. (D) HR-LCMS spectrum confirmed the annotation of structure as bohemamine B.

B

A

C

D

Figure S17. Smart 2.0 and HR-LCMS help with dereplication of **16.** (A) HSQC NMR of sample in CD_3_OD (400MHz, 298K). (B) Digitalized HSQC of the semipure **16**. (C) SMART 2.0 results (top 6 structures based on cosine similarity score) suggests annotation of structure as 5-chlorobohemamine C. (D) HR-LCMS spectrum confirmed the annotation of structure as 5-chlorobohemamine C.

E

Figure S18. Isotope pattern of 5-chlorobohemamine C **16.**


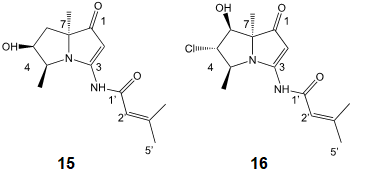


| No. | Bohemamine B  ^13^C ^1^H, mult. | | Compound 15  ^13^C ^1^H, mult | | 5-chloroBohemamine C  ^13^C ^1^H, mult | | Compound 16  ^13^C ^1^H, mult | |
| --- | --- | --- | --- | --- | --- | --- | --- | --- |
| 1 | 204.6 |  | 206.5 |  | 200.8 |  | 206.5 |  |
| 2 | 93.1 | 5.56(1H,s) | 93.1 | 5.59,(1H,s) | 94.9 | 5.55(1H,s) | 91.3 | 5.39(1H,s) |
| 3 | 167.7 |  | 164.7 |  | 166.8 |  | 163.4 |  |
| 4 | 57.9 | 3.86(1H,dq) | 55.2 | 3.92(1H,dq) | 63.8 | 4.07(1H,dq) | 57.5 | 4.02(1H,dq) |
| 5 | 72.2 | 4.44(1H,dddd) | 72.6 | 4.62(2H,dddd) | 70.9 | 4.21(1H,t) | 73.6 | 4.03(1H,t) |
| 6 | 35.6 | 1.55 dd,1.69 dd | 34.6 | 1.76 dd,1.93 dd | 78.9 | 3.99(1H,ddd) | 81.6 | 3.77(1H,ddd) |
| 7 | 69 |  | 69.6 |  | 76.9 |  | 76.9 |  |
| 4-Me | 9.8 | 0.9(3H,d) | 8.5 | 1.09(3H,d) | 17.0 | 1.32(3H,d) | 12.0 | 1.47(3H,d) |
| 7-Me | 26.1 | 1.13(3H,s) | 24.3 | 1.32(3H,s) | 24.4 | 1.41(3H,s) | 17.2 | 1.37(3H,s) |
| 1’ | 164 |  | 165 |  | 163.8 |  | 163.7 |  |
| 2’ | 117.6 | 5.98(1H,s) | 116 | 5.95(1H,s) | 117.5 | 5.95(1H,dq) | 116 | 5.86,dq |
| 3’ | 156.4 |  | 158.2 |  | 156.5 |  | 158.2 |  |
| 4’ | 27.3 | 1.88(3H,d) | 25.6 | 1.96(3H,s) | 27.3 | 1.89(3H,d) | 25.6 | 1.96(3H,d) |
| 5’ | 20 | 2.12(3H,d) | 18.4 | 2.22(3H,s) | 20.0 | 2.14(3H,d) | 18.4 | 2.22(3H,d) |

Table S6. Comparison of the ^1^H and ^13^C-NMR of **15 and 16** with the NMR data reported in literature for Bohemamine B and 5-chloroBohemamine C, respectively [10] (CD_3_OD, ^1^H-NMR at 400 MHz, ^13^C-NMR at 100 MHz)

A

B

C

D

Figure S19. Smart 2.0 and HR-LCMS help with dereplication of bohemamine (**17**). (A) HSQC NMR of sample in CD3OD (400MHz, 298K). (B) Digitalized HSQC of semipure **17**. (C) SMART 2.0 results (top 6 structures based on cosine similarity score) suggests annotation of structure as bohemamine. (D) HR-LCMS spectrum confirmed the annotation of structure as bohemamine.

A

B

C

D

Figure S20. Smart 2.0 and HR-LCMS help with dereplication of bohemamine F (**18**) (A) HSQC NMR of sample in CD_3_OD (400MHz, 298K). (B) Digitalized HSQC of semipure **18**. (C) SMART 2.0 results (top 6 structures based on cosine similarity score) suggests annotation of structure as bohemamine F. (D) HR-LCMS spectrum confirmed the annotation of structure as bohemamine F.

| No. | Bohemamine  ^13^C ^1^H, mult. | | Compound 17  ^13^C ^1^H, mult. | |  | Bohemamine F  ^13^C ^1^H, mult. | | Compound 18  ^13^C ^1^H, mult. | |
| --- | --- | --- | --- | --- | --- | --- | --- | --- | --- |
| 1 | 199.0 | - | 199.1 | - |  | 203.c |  | 195.6 |  |
| 2 | 91.7 | 5.34(1H,s) | 93.0 | 5.75(1H,s) |  | 93.1 | 5.58(1H,s) | 92.9 | 5.74(1H,s) |
| 3 | 168.6 | - | 168.5 | - |  | 168 |  | 169 |  |
| 4 | 55.5 | 3.8(1H,br,q) | 60.2 | 4.11(1H,br,q) |  | 62 | 4.40(1H,ddd) | 62.1 | 4.52(1H,ddd) |
| 5 | 56.7 | 3.58(2H,d) | 62.8 | 3.74(2H,d) |  | 128 | 5.74(1H,dd) | 127.6 | 5.83(1H,dd) |
| 6 | 56.4 | 3.58(2H,d) | 62.8 | 3.55(2H,d) |  | 133.9 | 5.67(1H,dd) | 134.0 | 5.69(1H,dd) |
| 7 | 72.8 | - | 72.6 | - |  | 80.2 | - | 81.4 | - |
| 4-Me | 13.9 | 1.20(3H,s) | 11.7 | 1.14(3H,s) |  | 24.8 | 1.22(3H,s) | 17.2 | 1.29(3H,s) |
| 7-Me | 18.8 | 1.35(3H,d) | 22.5 | 1.54(3H,d) |  | 17.9 | 1.19(3H,d) | 24.0 | 1.37(3H,d) |
| 1’ | 163.8 | - | 164.2 | -- |  | 164 | - | 169.5 | - |
| 2’ | 117.5 | 5.90(1H,s) | 117.4 | 5.85(1H,s) |  | 117.5 | 5.98(1H,m) | 117.1 | 5.86 |
| 3’ | 156.7 | - | 157.9 | - |  | 156.6 | - | 158.3 | - |
| 4’ | 27.3 | 1.87(3H,s) | 26.4 | 1.94(3H,s) |  | 27.3 | 1.9(3H,s) | 26.3 | 1.94(3H,s) |
| 5’ | 20.0 | 2.12(3H,s) | 18.9 | 2.20(3H,s) |  | 20 | 2.15(3H,s) | 19.1 | 2.22(3H,s) |

Table S7. Comparison of the ^1^H and ^13^C-NMR of **17 and 18** with the NMR data reported in literature for Bohemamine and Bohemamine F, respectively [11] (CD_3_OD, ^1^H-NMR at 400 MHz, ^13^C-NMR at 100 MHz)

| MIC (μg/mL) | compound **10** | Amp | Van |
| --- | --- | --- | --- |
| *Escherichia coli* ATCC 25922 | 3.11 | 4.1 | >50 |
| *Pseudomonas aeruginosa* ATCC 27853 | 13.2 | 0.6 | >50 |
| *Staphylococcus haemolyticus* | 36.2 | 3.3 | 14.8 |
| *S. epidermidis* ATCC 35984 | 11.4 | 3.5 | 0.02 |

Table S8. MIC value of tested compounds against a panel of pathogens

Figure S21. MIC curve of compound **10** against *Escherichia coli* ATCC 25922

| **Table S9** . Deduced functions of ORFs in *lga* biosynthetic gene cluster | | | | | |  |
| --- | --- | --- | --- | --- | --- | --- |
| **Gene** | **Size^a^** | **proposed function** | **protein homologue** | **%identity/%positives** | **Accession no.** | |
| *orf-1* | 384 | Hypothetical protein | EST92_RS03040a | 56%/55% | WP_129279411.1 |  |
| *lgaA* | 350 | C-methyltransferase | BhmG | 84%/92% | OKJ61996.1 |  |
| *lgaB* | 319 | Oxidoreductase | BhmH | 89%/95% | OKJ61997.1 |  |
| *lgaC* | 367 | Oxidoreductase | BhmI | 90%/95% | OKJ61998.1 |  |
| *lgaD* | 2054 | NRPS | BhmJ | 84%/89% | OKJ61999.1 |  |
| *lgaE* | 292 | FAD-dependent monooxygenase | BhmK | 93%/95% | OKJ62000.1 |  |
| *lgaF* | 373 | Branched-chain alpha-keto acid dehydrogenase | BhmLb | 94%/95% | OKJ62001.1 |  |
| *lgaG* | 262 | Acyl-CoA dehydrogenase | BhmO | 92%/94% | OKJ62003.1 |  |
| *lgaH* | 393 | TetR family transcriptional regulator | ADK54_RS40960 | 26%/36% | WP_053730951.1 |  |

^a^ Numbers are in Amino Acids

**Reference:**

1. Huang, S.; Tabudravu, J.; Elsayed, S.S.; Travert, J.; Peace, D.; Tong, M.H.; Kyeremeh, K.; Kelly, S.M.; Trembleau, L.; Ebel, R.; et al. Discovery of a single monooxygenase that catalyzes carbamate formation and ring contraction in the biosynthesis of the legonmycins. *Angew. Chemie - Int. Ed.* **2015**, *54*, 12697–12701.

2. Johnston, C.W.; Zvanych, R.; Khyzha, N.; Magarvey, N.A. Nonribosomal Assembly of Natural Lipocyclocarbamate Lipoprotein-Associated Phospholipase Inhibitors. *ChemBioChem* **2013**, *14*, 431–435.

3. Schimming, O.; Challinor, V.L.; Tobias, N.J.; Adihou, H.; Grün, P.; Pöschel, L.; Richter, C.; Schwalbe, H.; Bode, H.B. Structure, biosynthesis, and occurrence of bacterial pyrrolizidine alkaloids. *Angew. Chemie - Int. Ed.* **2015**, *54*, 12702–12705.

4. Hong, Z.; Bolard, A.; Giraud, C.; Prévost, S.; Genta‐Jouve, G.; Deregnaucourt, C.; Häussler, S.; Jeannot, K.; Li, Y. Azetidine‐Containing Alkaloids Produced by a Quorum‐Sensing Regulated Nonribosomal Peptide Synthetase Pathway in *Pseudomonas aeruginosa*. *Angew. Chemie* **2019**, *131*, 3210–3214.

5. Liu, L.; Li, S.; Sun, R.; Qin, X.; Ju, J.; Zhang, C.; Duan, Y.; Duan, Y.; Duan, Y.; Huang, Y.; et al. Activation and Characterization of Bohemamine Biosynthetic Gene Cluster from *Streptomyces* sp. CB02009. *Org. Lett.* **2020**, *22*, 4614–4619.

6. Qin, Z.; Devine, R.; Booth, T.J.; Farrar, E.H.E.; Grayson, M.N.; Hutchings, M.I.; Wilkinson, B. Formicamycin biosynthesis involves a unique reductive ring contraction. *Chem. Sci.* **2020**, *11*, 8125–8131.

7. Beam, M.P.; Bosserman, M.A.; Noinaj, N.; Wehenkel, M.; Rohr, J. Crystal structure of Baeyer-Villiger monooxygenase MtmOIV, the key enzyme of the mithramycin biosynthetic pathway. *Biochemistry* **2009**, *48*, 4476–4487.

8. Wakimoto, T.; Egami, Y.; Nakashima, Y.; Wakimoto, Y.; Mori, T.; Awakawa, T.; Ito, T.; Kenmoku, H.; Asakawa, Y.; Piel, J.; et al. Calyculin biogenesis from a pyrophosphate protoxin produced by a sponge symbiont. *Nat. Chem. Biol.* **2014**, *10*, 648–655.

9. Zhang, Q.; K, K.S.; Elsohly, H.N.; Takamatsu, S. New Cell-cell Adhesion Inhibitors from *Streptomyces* sp. UMA-044. *J. Antibiot. (Tokyo).* **2003**, *56*, 673–681.

10. Bugni, T.S.; Woolery, M.; Kauffman, C.A.; Jensen, P.R.; Fenical, W. Bohemamines from a marine-derived *Streptomyces* sp. *J. Nat. Prod.* **2006**, *69*, 1626–1628.

11. Fu, P.; La, S.; MacMillan, J.B. 1,3-Oxazin-6-one Derivatives and Bohemamine-Type Pyrrolizidine Alkaloids from a Marine-Derived *Streptomyces spinoverrucosus*. *J. Nat. Prod.* **2016**, *79*, 455–462.
